# MINOCA as the result of coronary artery aneurysm thrombosis

**DOI:** 10.1177/03000605241301859

**Published:** 2024-12-11

**Authors:** Oksana Rokyta

**Affiliations:** 1 Department of Internal Medicine No. 2, Bogomolets National Medical University, Kyiv, Ukraine

**Keywords:** MINOCA, coronary artery aneurysm, coronary artery thrombosis, aetiology of coronary artery aneurysm, congenital coronary artery aneurysm

## Abstract

Myocardial infarction (MI) can be caused by many factors. In addition to the typical obstruction or stenosis of the coronary arteries, there is heterogenic MI with non-obstructive coronary arteries (MINOCA). A rare cause of MINOCA is the thrombosis of a coronary artery aneurysm (CAA). This current case report describes a male patient with CAA thrombosis as the cause of MINOCA following surgery for a mucoepidermoid carcinoma. The patient underwent angiography that identified three CAAs that were located as follows: (i) in the proximal part of the left anterior descending artery (5.55 mm); (ii) in the distal part of the circumflex artery (8.05 mm); and (iii) in the distal part of the right coronary artery (6.61 mm). Thrombotic masses were identified within all three structures. The patient received balloon angioplasties without stent implanting and recovered well. The patient was also notable for the presence of two brain artery aneurysms that were the cause of the previous strokes that he had experienced. This case report also reviews the literature in order to: (i) summarize the aetiological factors and clinical manifestations of CAA; (ii) discuss the diagnostic methods for CAA; (iii) describe the medical and surgical management of CAA; and (iv) assess the prognosis of this rare clinical event.

## Introduction

Myocardial infarction (MI) remains a significant concern in modern cardiology. In 2020, cardiovascular diseases were responsible for approximately 19 million deaths worldwide, which was an 18.7% increase from 2010.^1^ Pathologically, MI is defined as the death of myocardial cells resulting from prolonged ischaemia.^2^ While atherosclerosis and rupture of artery plaque are the primary causes of acute coronary syndrome (ACS) in most patients, non-obstructive processes also play a role in a substantial number of ACS cases. Therefore, different diagnostic and management strategies are required.^2^ This current clinical case highlights coronary artery aneurysm thrombosis as one of the causes of MI with non-obstructive coronary arteries (MINOCA). Due to its rare incidence, the heterogeneous origins of this vascular pathology have not been extensively investigated.^3,4^ A deeper understanding of such causes is essential for effective treatment and prevention. MINOCA accounts for 5–6% of all MI, with the prevalence varying from 5% to 15% depending on the population being examined.^4^ Findings from a large observational study involving 286 780 patients with MINOCA revealed a 12-month frequency of 18.7% of major adverse cardiovascular events (MACE), including mortality, MI, heart failure and stroke.^5^ Non-ST elevation MI (NSTEMI) has been reported in the majority of MINOCA cases (two-thirds), while the rest present with suspected ST elevation MI (STEMI).^4^

**Figure 1.**
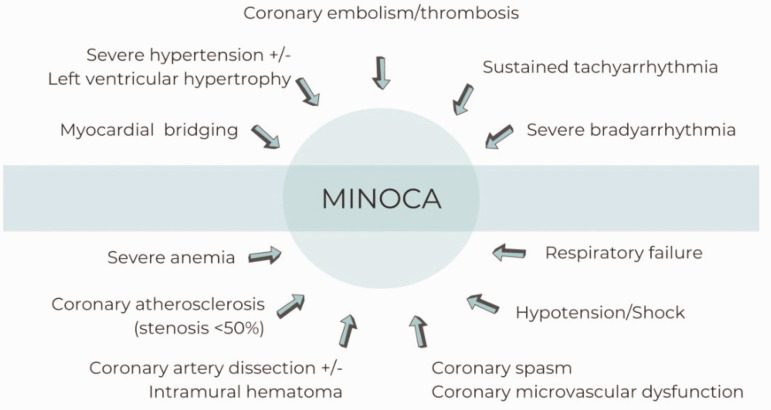
The causes of myocardial infarction with non-obstructive coronary arteries (MINOCA).

The diagnostic criteria for MINOCA include the following: (i) evidence of MI according to the Fourth Universal Definition of Myocardial Infarction;^2^ (ii) presence of non-obstructive changes of coronary arteries on angiography (no coronary artery stenosis in any major epicardial vessel); (iii) absence of an alternate diagnosis, such as sepsis, pulmonary embolism, or myocarditis.^4^ MINOCA can manifest itself as either MI type 1 (no obstructive atherosclerotic plaque disruption) or MI type 2 (misbalance of supply and demand).^6^ The causes of MINOCA are highly heterogeneous and may include vascular pathology, arrhythmia, haemodynamic changes, haematological disorders and structural abnormalities (Figure 1).

In this current case report and review, particular attention is given to thrombosis as a cause of MINOCA, as well as rare vessel abnormalities such as coronary artery aneurysm (CAA). It is suggested that coronary thrombosis can lead to MINOCA either by affecting the microcirculation or due to partial lysis of non-obstructive epicardial coronary thrombus.^4^ Hypercoagulation is identified as the primary cause of thrombosis in MINOCA patients.^4,5^ Acquired hypercoagulable disorders include thrombotic thrombocytopenic purpura,^7^ the autoimmune disorder antiphospholipid syndrome,^8,9^ heparin-induced thrombocytopenia and myeloproliferative neoplasms.^10^ CAA, which is characterized by abnormal vessel dilatation, creates conditions conducive to thrombus formation due to stagnant blood flow and reduced shear stress.^11^ CAA-related thrombosis has been identified as the cause of ACS in several studies.^12–15^

## Case report

In October 2021, a male patient in his early 60 s was admitted to the Cardiac Intensive Care Unit, Oleksandrivska Kyiv City Clinical Hospital, Bogomolets National Medical University, Kyiv, Ukraine presenting with retrosternal burning pain and general weakness. He had not previously experienced angina. He had received surgery for cancer 4 days previously. The physical examination showed the following: temperature, 36.8 °C; blood pressure, 150/90 mmHg; heart rate, 88 beats per min; respiratory rate, 20 breaths per min; oxygen saturation, 97% when breathing room air. His cardiovascular examination was significant only for weak S1, but there were no audible murmurs, rubs or gallops. His lungs were clear and no peripheral oedema was observed. An irregular sinus rhythm was registered on an electrocardiogram (ECG), which was attributed to ventricular premature complexes, along with an incomplete right bundle branch block (RBBB). ST elevation was noted in leads I, aVL and V5–V6. The troponin I level was positive at 25.61 ng/ml. The patient was diagnosed lateral STEMI.

**Figure 2.**
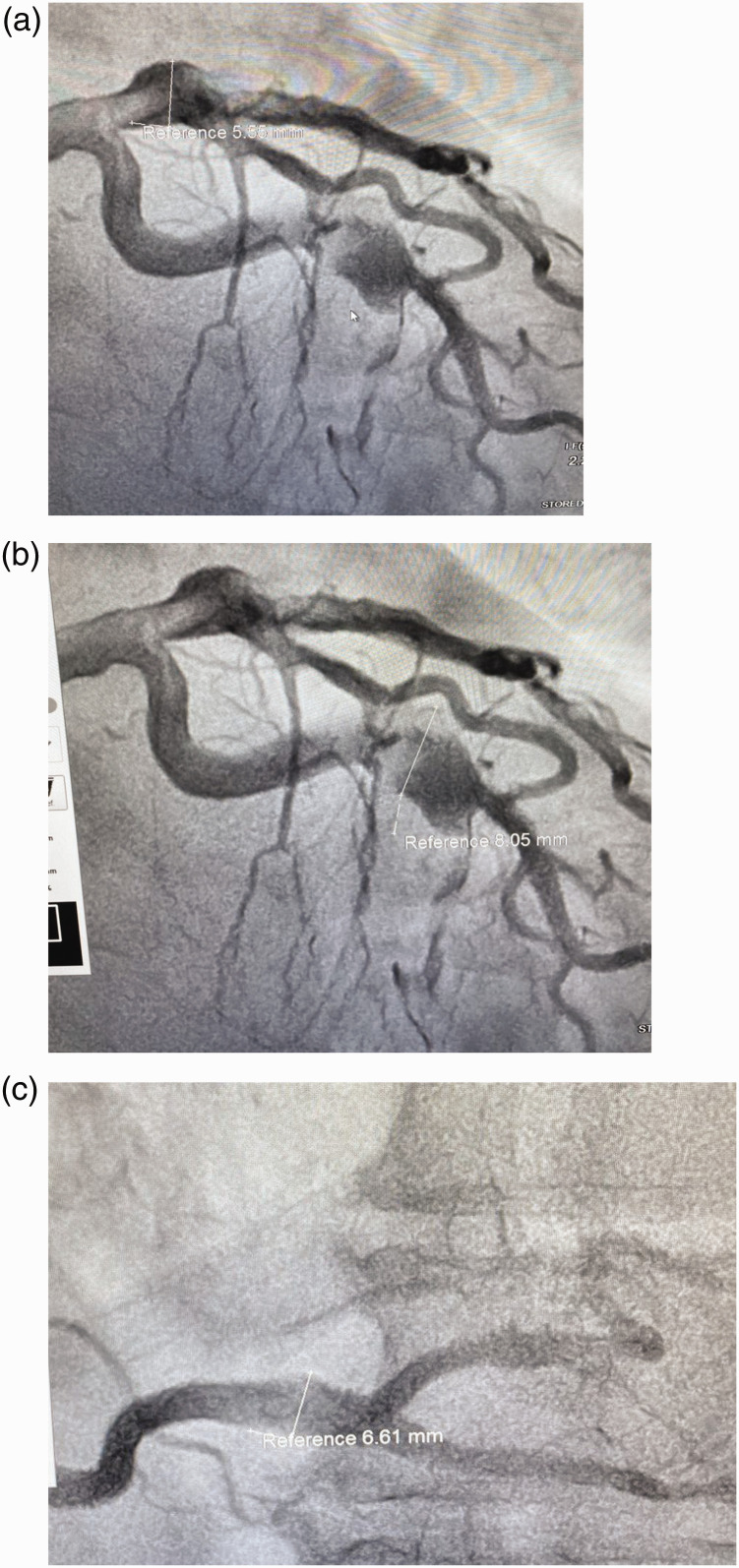
Coronary angiography images of a male patient in his early 60s who presented with retrosternal burning pain and general weakness who was subsequently diagnosed with myocardial infarction with non-obstructive coronary arteries (MINOCA). Coronary artery aneurysms were identified as follows: (a) in the proximal part of the left anterior descending artery with an aneurysm transverse size measuring 5.55 mm; (b) in the distal part of the circumflex artery measuring 8.05 mm; and (c) in the distal part of the right coronary artery measuring 6.61 mm.

The patient underwent angiography (Figure 2), revealing uncommon vascular changes. These changes manifested as three CAAs, specifically located as follows: (i) in the proximal part of the left anterior descending (LAD) artery with an aneurysm transverse size measuring 5.55 mm; (ii) in the distal part of the circumflex artery measuring 8.05 mm; and (iii) in the distal part of the right coronary artery (RCA) measuring 6.61 mm. Thrombotic masses were identified within all of these structures. Balloon angioplasties were undertaken without stent implanting. The patient’s echocardiogram after angiography revealed normal sizes of the heart chambers, but hypertrophy of the left ventricular (LV) wall (1.25 mm), no hypokinesis and preserved ejection fraction of the LV of 57%. On the 8th day after the MI, the ECG showed sinus rhythm, RBBB, the presence of Q wave and negative T-wave (–) in leads I, aVL and V5–V6.

At hospital admission (05.10.2021), the patient had significantly elevated C-reactive protein (CRP) and evidence of hypercoagulation (Table 1), but there was no evidence of hyperlipidaemia. These laboratory findings could have resulted from his oncological disease. The hypercoagulation, typically associated with oncological pathologies, might have contributed to CAA thrombosis. In July 2021, the patient was diagnosed with mucoepidermoid carcinoma. This is an uncommon malignant salivary gland tumour, which accounts for 10–15% of all salivary gland tumours.^16^ The results of the patient’s histological investigation indicated mucoepidermoid carcinoma with a predominance of mucin-secreting cells, along with partial paravessel and paraneural invasion. Researchers have found that most mucoepidermoid carcinomas have the *MECT1-MAML2* fusion oncogene.^17^ A complete tumour resection with negative surgical margins and lymph node dissection were performed on 1 October 2021 and the STEMI occurred on 4 October 2021.

**Table 1.** Laboratory data for a male patient in his early 60s who presented with retrosternal burning pain and general weakness who was subsequently diagnosed with myocardial infarction with non-obstructive coronary arteries.

Complete blood count 04.10.2021	Red blood cells: 4.9 × 10^12^/l								
	White blood cells: 11.5 × 10^9^/l								
	Haemoglobin: 148 g/l								
	Haematocrit: 44.3%								
	Platelet count: 286 × 10^9^/l								
	Erythrocyte sedimentation rate: 40 mm/h								

Coagulogram 04.10.2021	aPTT: 17 s								
	Thrombin time: 10 s								
	Fibrinogen: 56 g/l								

Biochemical analyses	Creatinine, µmol/l	Total protein, g/l	Albumin, g/l	ALT, U/l	AST, U/l	CRP, mg/l	Glucose, mmol/l	LDL-C, mmol/l	Total cholesterol, mmol/l

05.10.2021	84	69	42	33	140	150	6.2		
07.10.2021	105			41	57		HbA1c 5.5%	1.6	3.6
14.10.2021	75					15			

aPTT, activated partial thromboplastin time; ALT, alanine aminotransferase; AST, aspartate aminotransferase; CRP, C-reactive protein; LDL-C, low-density lipoprotein cholesterol; HbA1c, glycosylated haemoglobin.

The patient’s medical history was also notable for the presence of other vessel aneurysms. Twice, in 2014 and 2018, the patient experienced a stroke. In 2018, angiography was conducted to diagnose and treat the disturbance of brain blood supply. The results showed the presence of a fusiform aneurysm in the basilar artery (with a diameter of 6.6 mm) and an aneurysm in the left vertebral artery (with a diameter of 6.2 mm) that exerted pressure on the medulla. No stenosis of the brain arteries was observed. Stent implantation was recommended, but the patient refused to undergo the procedure.

Before the current discharge, to rule out other arterial aneurysms, a computer tomography angiography of the thoracic, abdominal aorta and renal arteries was performed. No additional malformations were observed. According to these current data and the absence of the main risk factors (such as dyslipidaemia, obesity, diabetes mellitus, smoking), atherosclerosis was excluded. Vasculitis was suspected to be the cause of CAA in this current case due to high level of the inflammatory markers and multivessel pathology. Initially, Kawasaki disease had to be excluded, because such pathology is the main cause of CAA among vasculatis.^18^ The important diagnostic criteria (i.e. young age, fever, rash, lymphadenopathy) were absent in the current patient. CAAs are observed in patients with polyarteritis nodosa as result of inflammation of medium size arteries.^19^ The current patient had elevated erythrocyte sedimentation rate and CRP, but he did not report weight loss, abdominal and testicular pain. A physical examination revealed no skin nodules or mononeuritis multiplex. Hepatitis B serologies were also negative.

The coexistence CAA and salivatory gland enlargement can be the criteria of immunoglobulin (Ig)G4-related disease, which is a systemic fibroinflammatory disease characterized by dense infiltration of IgG4-positive plasma cells in the affected tissue(s) with or without elevated plasma levels of IgG4.^20^ The current patient had normal levels of IgG4 and there was no evidence of the typical changes in the thyroid and pancreas. The histological analysis of a salivary gland biopsy detected mucin-secreting cell infiltration. CAAs develop in 10–30% of the patients with Takayasu’s arteritis.^18^ Coronary pathology also includes ostial stenosis and skip lesions. This diagnosis was excluded in the current patient due to normal aorta and subclavian artery sizes on the angiographic investigation. The patient was also examined for the presence of HIV, syphilis and mycobacterial infections, which were all negative. The patient denied drug abuse.

Taking all of the findings into account, the most likely aetiology of current patient’s coronary and brain aneurysms was genetic susceptibility. Finally, he was diagnosed with the following: CAD; MINOCA: acute Q lateral myocardial infarction (4.10.21); coronary artery aneurysms; arterial hypertension II stage; heart failure with preserved ejection fraction (57%); stroke (2014, 2018); brain artery aneurysms; right salivatory gland resection (1.10.21) due to mucoepidermoid carcinoma.

After receiving the angiography, the patient was administered enoxaparin 0.4 ml intravenous twice a day, 90 mg ticagrelor oral twice a day and 75 mg aspirin oral once a day despite the high risk of bleeding in the early postoperative period. Additional medical management included: 2.5 mg bisoprolol oral once a day, 2.5 mg ramipril oral once a day and 20 mg atorvastatin oral once a day. The patient was recommended to continue the same dosages of aspirin, ticagrelor, bisoprolol, ramipril and atorvastatin for 1 year. He was also advised to be remain under the care of a cardiologist and an oncologist. Patient consent was provided for treatment. The author has de-identified all patient details. The reporting of this case report conforms to the CARE guidelines.^21^

## Discussion

A wide range of CAA frequencies, varying from 0.3% to 5.3%, has been observed depending on inclusion criteria.^22^ The CAA register confirmed a low frequency of 0.35%.^23^ A comprehensive review spanning from 2004 to 2016, involving a large cohort of over 1500 patients with CAA from 32 hospitals worldwide (in nine countries), was conducted.^23^

Several studies have assessed the outcomes of CAA, particularly its impact on mortality. For example, CAA was identified as an independent predictor of death, with an overall 5-year survival rate of 71% for CAA patients.^24^ Data from a prospective study lasting 52 months revealed a 12.8% mortality rate (26.4% of which were cardiac causes) and a 42% incidence of cardiovascular events.^25^ The presence of CAA was deemed an independent risk factor for both mortality (hazard ratio 3.1; 95% confidence interval [CI] 1.8, 5.6; *P* < 0.001) and MACE (hazard ratio 2.3; 95% CI 95% 1.4, 3.8; *P* < 0.001), as calculated using various Cox multivariate models.^25^ According to the Coronary Artery Aneurysm Registry (CAAR), mortality and MACE rates were 15.3% and 31%, respectively.^23^

Different types of CAA are distinguished as follows: saccular aneurysm (longitudinal diameter < transverse diameter); fusiform aneurysm (longitudinal diameter > transverse diameter); coronary artery ectasia (diffuse enlargement > 1.5 times the adjacent normal segment); and pseudoaneurysm loss of vessel wall integrity.^26–28^ There are conflicting data regarding the frequency of aneurysms in each coronary artery.^23,25,26,28,29^ According to the more representative CAAR study, aneurysms are predominantly located in the LAD artery (48.6%), followed by the RCA (31.8%) and the circumflex (28.1%).^23^ Aneurysms were also observed in the left main artery. Similar results were demonstrated in other studies,^25,29^ although some studies indicate a higher prevalence in the RCA.^26,28^ Isolated CAA were more frequently observed (83%), followed by both CAAs (12.8%).^23^ The variation in CAA frequency is attributed to the highly heterogeneous nature of this pathology, encompassing both hereditary and acquired as well as iatrogenic etiological factors (Table 2).^13,18,27,29–40^

**Table 2.** The aetiology of coronary artery aneurysm (CAA).^13,18,27,29–40^

Cause	Frequency	Vessel changes/pathogenesis
Atherosclerosis	50%^27^	Vessel changes (lipid deposit, inflammation, focal fibrosis, calcification of tunica media) resulted in decreased intraluminal pressure tolerance that leads to vessel dilatation.^27^
		Activity of matrix metalloproteinases (MMPs) plays a role in proteolysis of connective tissue proteins. The *MMP-3A* allele was associated with the occurrence of coronary artery aneurysm in an older population (mean age 62 years).^30^
Congenital	17–30%^27^	The vessel exhibits changes, including a thinner tunica media and replacement of the tunica media with hyalinized tissue. Additionally, there was focal calcification and loose connective tissue containing some arterioles and venules. Perivascular infiltration of lymphocytes was also observed.^27^
		Disruption of the *HLA-E* and *MMP-3* genes, insertion/deletion polymorphisms in the angiotensin-converting enzyme (ACE DD genotype) and variations in the *SRC-1* and *GRIN3A* genes resulted in the thinning of the vessel wall.^31^
		Specific HLA class II genotypes: HLA-DR B1*13, DR16, DQ2 and DQ5.^13,27^
Kawasaki disease	≈10%^27^ 17%^29^	Inflammatory vessel changes (infiltration of arterial wall [mononuclear, lymphocytes, and macrophages]); necrosis of smooth muscle cells; myointimal proliferation.^31^
		High level of the inflammatory cytokine tumour necrosis factor alpha. Gene mutations in the intron of the *TIAM1* gene resulted in stimulating chemokine-induced T cell migration and infiltration of lymphocytes into the vascular wall by TIAM1 protein.^32^
		Variation in *MMP-9* gene polymorphisms.^30^
Polyarteritis nodosa	30–50% (incidence of CAA among the patients with polyarteritis nodosa)^18^	Distinct vessel alterations were observed, which were characterized by focal panmural necrotizing inflammatory lesions, fibrinoid necrosis and infiltration primarily consisting of polymorphonuclear leukocytes.^27^
Takayasu arteritis	10–30% (incidence of CAA among the patients with Takayasu arteritis)^18^	Resulting from the destruction of elastic fibres in the tunica media and the thickening of the tunica adventitia, media and intima.^27^
Behçet’s disease	0.5–2% (incidence of CAA among the patients with Behçet’s disease)^18^	Inflammatory alterations in the vessel include endothelial cell swelling and mononuclear perivascular infiltration, leading to obliterative endarteritis of the vasa vasorum, resulting in the destruction of the tunica media and fibrosis.^27^
Immunoglobulin G4-related disease	1–3% (incidence of CAA among the patients with IgG4)^18^	Non-specific.^27^
Other forms of vasculitis include those associated with conditions such as lupus, rheumatoid arthritis, ankylosing spondylitis, scleroderma and others	Rare	Non-specific.^27^
Connective tissue diseases encompass a range of conditions, including Marfan syndrome, Ehlers-Danlos syndrome, fibromuscular dysplasia, neurofibromatosis and others	Rare	Vessel changes associated with cystic degeneration involve the disruption of connective tissue elements and the accumulation of acid mucopolysaccharides in the tunica media, and occasionally in the tunica intima.^27^ Overactivity of transforming growth factor beta is implicated in cystic medial necrosis.^33^
Infections, including HIV, bacterial infections, mycobacterial infections, syphilis, Lyme disease, mycotic aneurysm and septic emboli		Direct damage of the vessel wall by microorganisms or immune complex deposition.^34^
Drugs such as cocaine, amphetamine and protease inhibitors		Dynamic wall stress changes, episodic hypertension and vasoconstriction can contribute to endothelial damage.^35^
Percutaneous coronary intervention-associated Type I aneurysm demonstrating rapid early growth with pseudoaneurysm formation detected within 4 weeksType II aneurysms are typically detected incidentally during angiography performed for recurrent symptoms or as part of protocol-mandated follow-upType III aneurysms are mycotic or infectious in aetiology^36^	1.25–3.9%^37^	Coronary dissection and last stent malapposition.^38^
		Vasculitis leading to eosinophilic or heterophilic infiltration into the vessel wall can occur as a result of drug-eluting stent implantation.^39^
		Decreased neointimal formation, accompanied by persistent fibrin deposition and a macrophage infiltration response to drug-eluting stent.^40^

Coronary artery aneurisms do not exhibit specific clinical features and their manifestation as stable angina is the most common presentation.^23^ In addition, patients may present with STEMI or NSTEMI, syncope, sudden cardiac death, fistula formation, rupture, haemopericardium, tamponade, compression of surrounding structures or congestive cardiac failure.^25,26,41^

Acute coronary syndrome in CAAs is often attributed to thrombi formation on the irregular internal surface of the aneurysm or distal embolization. The highest risk of thrombi occurrence is noted when the diameter of the aneurysm exceeds 5 mm.^42^ Patients may manifest with STEMI, NSTEMI or sudden cardiac death.^23,25^ Aneurysm rupture with subsequent heart tamponade is a catastrophic complication of CAA.^41^ In some studies, CAAs have been linked to the development of chronic heart failure.^23,27^ Compression of the surrounding structures is a common manifestation of giant CAAs.^27^ Patients may exhibit symptoms of superior vena cava syndrome. The differential diagnoses for giant CAAs include cysts, cardiac tumours and other masses.^43^

Aneurysm formation may extend beyond coronary arteries and involve other vascular systems. A review of the published literature to identify cases with coexisting CAA and aneurysms at multiple locations identified 61 articles with a total of 76 patients (mean ± SD age: 37.4  ±  26.5 years; male: 58 [76.3%]).^44^ According to their results, the most common locations of concomitant vascular aneurysms were the abdominal aorta (*n*  = 40; 52.6%) and the common iliac artery (*n* = 38; 50.0%).^44^ Other sites of concomitant vascular aneurysms included all medium-sized arteries.^44^

## Diagnosis of CAAs

Various imaging techniques used to diagnose CAA include coronary angiography, intravascular ultrasound (IVUS), computed tomography angiography (CT angiography), coronary magnetic resonance angiography (MRA) and echocardiography. Coronary angiography remains the most commonly employed method for identifying CAA. It enables the assessment of location, shape and frequency of aneurysms. Coronary angiography provides information about thrombosis presence, coronary artery stenosis and the extent of collateral artery formation.^45,46^ IVUS is considered the ‘gold standard’ for diagnosing CAA. It allows for the evaluation of the arterial wall structure and luminal composition, facilitating differentiation between various types of aneurysms.^47^ Non-invasive methods are becoming more widely used for the diagnosis of CAA. CT angiography offers a three-dimensional evaluation of CAA morphology, including maximum diameter and shape, vessel wall composition, presence of thrombosis and concomitant stenosis, plaque composition and the location of CAA in relation to the surrounding vasculature.^48^ Coronary MRA is useful for diagnosing CAAs in proximal coronary artery segments, providing information about flow rate and character.^49^ However, smaller distal segments remain invisible. Echocardiography is useful for diagnosing CAAs, especially in cases of Kawasaki disease in children.^13^

## Management of CAAs

Currently, there are no guidelines for the diagnosis and management of patients with CAA. Recommendations are derived from small-scale research studies, given the rarity of this pathology and the heterogeneity of its aetiology.^13,23,25,28^

## Medical management of CAAs

Medical therapy for CAAs encompasses the use of antithrombotic/anticoagulant medications and risk modification therapy.^26,50,51^ As atherosclerosis is considered the main aetiology for CAAs, single or dual antiplatelet therapy is recommended in some research studies.^50,51^ It is suggested as the option for the asymptomatic patient with CAA.^28^ Antiplatelet medications were widely prescribed in the CAAR, with aspirin being the most commonly prescribed (90.2%).^23^ Dual therapy, with a duration of 12 months, was utilized in 64.8% of cases.^23^ For aneurysms with multivessel disease or in cases of high thrombotic risk, longer dual therapy or a combination with anticoagulants was proposed (13.4%).^23^

Anticoagulants, specifically vitamin-K antagonists, are used in cases of coronary artery ectasia. A previous study demonstrated a potent beneficial effect of warfarin in such patients.^52^ Warfarin is also recommended for patients with Kawasaki disease, particularly those with large or rapidly expanding CAAs.^53^ However, according to the CAAR conclusions, aneurysms with multivessel disease or those occurring in the presence of other prothrombotic comorbidities could benefit from anticoagulation treatment.^23^

Matrix metalloproteinases (MMPs), which have been implicated in the increased proteolysis of extracellular matrix proteins, are considered part of the pathogenesis of CAA formation.^33^ This is why statins, known for their ability to inhibit MMP1 and MMP2, are proposed for the treatment of CAA.^54^ Angiotensin-converting enzyme inhibitors may also be prescribed for the same purpose.^55^ Intravenous immunoglobulin therapy is used in patients with Kawasaki disease for the treatment of CAAs.^53^ CAA with an autoimmune aetiology should be treated with immunosuppressive therapy according to the main disease. Glucocorticoids, cyclophosphamide and methotrexate are recommended for the management polyarteritis nodosa;^56^ with glucocorticoids and rituximab being recommended for IgG4-related disease.^20^

## Surgical treatment of CAAs

Surgical approaches include percutaneous coronary intervention (PCI) or surgical management. The choice depends on the clinical manifestation (asymptomatic, stable angina, MI, rupture, external compression) and CAA size/phenotype. The types of interventional therapy and the indications for their use are described in Table 3.^26–28,57–60^

**Table 3.** The types of interventional therapy for coronary artery aneurysm (CAA).^26–28,57–60^

	Percutaneous coronary intervention	Surgery
Procedure	Stent implantation	Resection of the aneurysm
		Proximal and/or distal ligation
	Drug-eluting stents	Aneurysmal thrombectomy
	Polytetrafluoroethylene stent graft	Marsupialization with interposition graft
	Coil embolization^28,57,58^	Associated with CABG^59,60^
Indication	Single-vessel or focal multivessel disease	CAAs in the left main stem
		CAAs near the bifurcation of large branches
		CAA complicated by fistula formation
	No left main coronary artery involvement	Compression of cardiac chambers
	No mechanical complications	Giant CAA (dilatation exceeding the reference vessel diameter by > four-times)
	Suitable anatomy for percutaneous coronary intervention	CAA complicated by embolization of distal part of coronary artery
	Acute coronary syndrome^27^	CAA progressive enlargement^26^

## Conclusion

In conclusion, despite the absence of significant coronary artery stenosis in the patient with MINOCA, such pathology can lead to STEMI and MACE. There are many different causes of MINOCA. The current case was diagnosed with MINOCA caused by the thrombosis of CAA. The aetiology of CAA includes atherosclerosis, congenital changes, vasculitis, connective tissue diseases, infections, drugs and PCI complications. The early diagnosis of artery aneurysms, especially silent aneurysms, prevents live-threatening complications such as MI, stroke and bleeding. Future research into the genetic predisposition for CAA should improve the timely diagnosis of this rare vascular pathology.
